# Supplementary material for: Rare mutations in the complement regulatory gene CSMD1 are associated with male and female infertility
Source: Nat Commun. 2019 Oct 11;10:4626. doi: 10.1038/s41467-019-12522-w (PMC6789153; doi:10.1038/s41467-019-12522-w)
Supplement: Supplementary file 1 — Supplementary Information [file 41467_2019_12522_MOESM1_ESM.pdf]

Supplementary information

Rare mutations in the complement regulatory gene CSMD1 are associated with male and female infertility

AS Lee *et al.*

Description of Supplementary Information items.

Supplementary Figures

Supplementary Fig. 1. Validation of *CSMD1* CNVs.

Supplementary Fig. 2. CSMD1 expression in human tissues.

Supplementary Fig. 3. Separating whole tissue into purified germ cell populations and validating Csmd1 expression patterns.

Supplementary Fig. 4. Documenting the effect of *Csmd1*$^{tm1Lex/tm1Lex}$ on RNA and protein expression in gonads and mammaries.

Supplementary Fig. 5. Detailed pathologic characterization of *Csmd1* knockout testes.

Supplementary Fig. 6. Male and female sex hormone measurements.

Supplementary Fig. 7. C3 deposition in Csmd1 wildtype versus knockout testes.

Supplementary Fig. 8. *C3*$^{-/-}$ gonads are histologically normal.

Supplementary Fig. 9. Complement deficiency unmasks severe morphological derangement in *Csmd1* knockout ovaries.

Supplementary Fig. 10. Functional genomic annotations of *CSMD1* indicate regulatory power of introns 1 and 2.

Supplementary Tables

Supplementary Table 1. Rare CNVs associated with gonadal function in WHI-SHARe and GEMINI.

Supplementary Table 2. Genotype-phenotype information for a subset of *CSMD1* 5'-deletion early idiopathic menopause cases in the WHI.

Supplementary Table 3. Results of breeding *Csmd1*$^{-/-}$; *C3*$^{-/-}$ double knockouts.

Supplementary References

Supplementary Figures

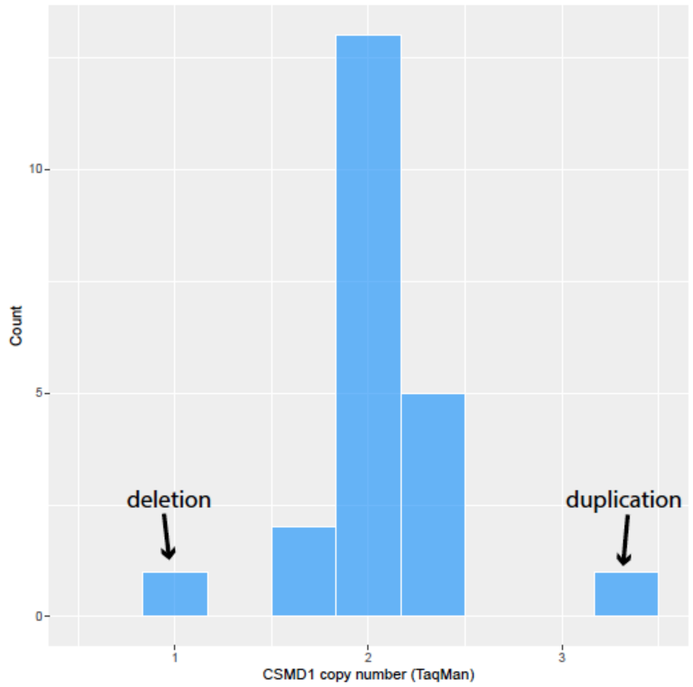

Supplementary Fig. 1. Validation of CSMD1 CNVs.
Histogram depicting estimated CSMD1 copy number from TaqMan assay. TaqMan copy number estimates and SNP array CNV calls were 100% concordant (22/22 samples). Source data are provided as a Source Data file.

A

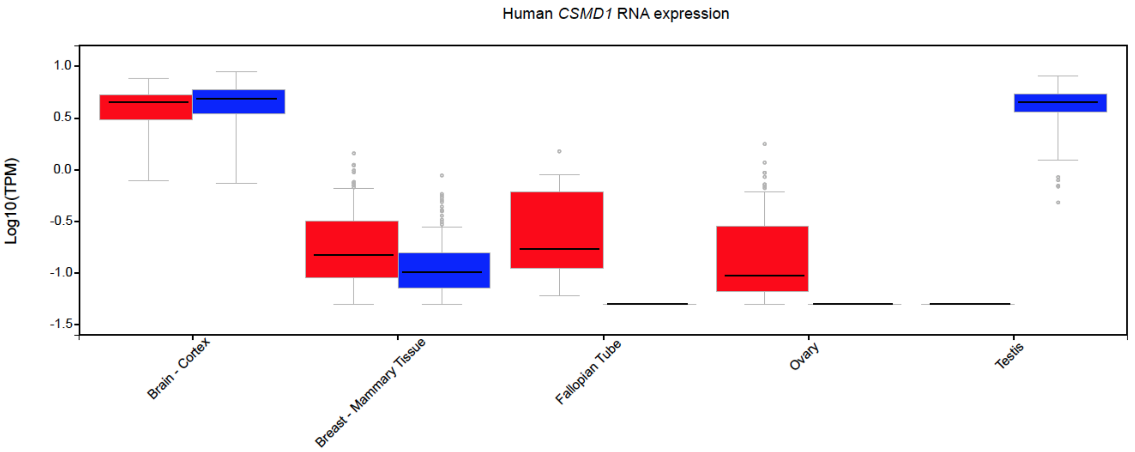

B

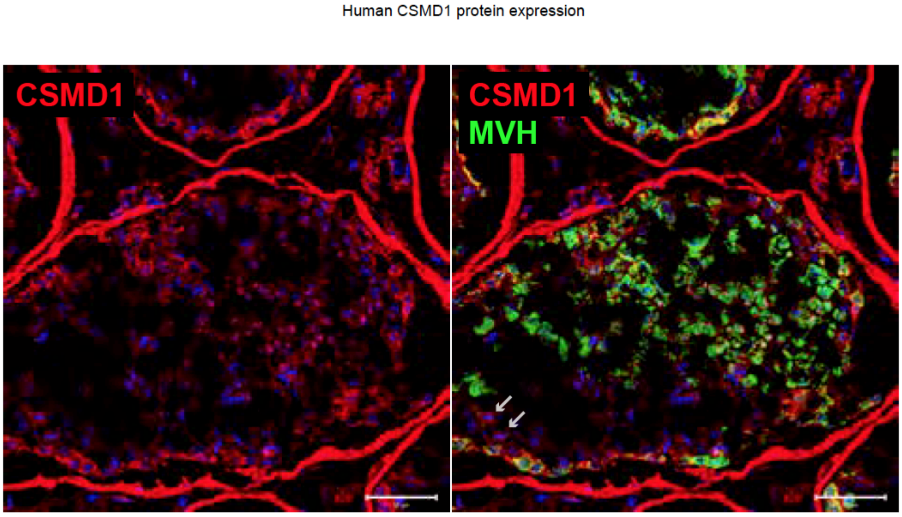

Supplementary Fig. 2. CSMD1 expression in human tissues.
(**A**) Modified screenshot from the GTEx portal (https://www.gtexportal.org/) depicting human *CSMD1* mRNA expression in brain, mammary, fallopian tube, ovary, and testis. Expression levels are stratified by sex (female = red, male = blue). (**B**) Immunofluorescence (IF) of CSMD1 protein (red) and early germ cell marker MVH (green) in human donor testis. CSMD1 protein signal is positive along MVH-negative hook-shaped elongating spermatids (white arrows), corroborating mouse RNA-seq and immunofluorescence data in **Fig. 2**.

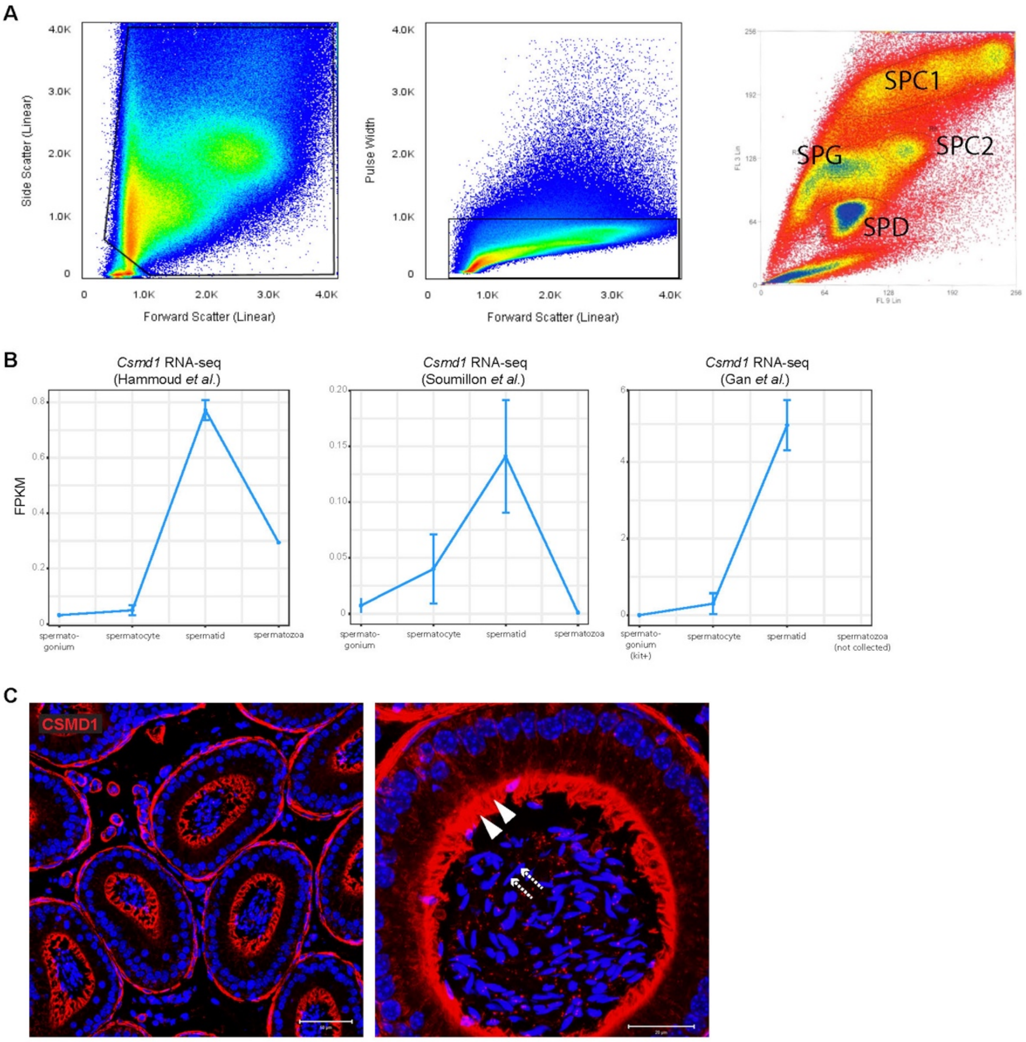

Supplementary Fig. 3. Separating whole tissue into purified germ cell populations and validating *Csmd1* expression patterns.

(**A**) Sequential gating strategies (left-to-right: side scatter vs. forward scatter, forward scatter vs. pulse width, and red vs. blue fluorescence) depicting stereotyped clustering of dissociated germ cells when stained with Hoechst. Due to the size and ploidy changes that accompany normal spermatogenesis, germ cells cluster along the red/blue spectra in a predictable and reproducible manner. Clusters corresponding to spermatogonia, primary spermatocytes, secondary spermatocytes, and spermatids are marked by "SPC", "SPC1", "SPC2", and "SPD", respectively. These purified germ cells were collected from Csmd1 WT and KO testes and sequenced as shown in Figures 2, 3, and Supplementary Fig. 4. (**B**) RNA expression of Csmd1 in FPKM of purified spermatogonia, spermatocytes, spermatids, and spermatozoa from previously published resources[1–3]. The germ cells from these previous studies were purified using orthogonal technologies to our cytometry-based method shown in (**A**) (enzymatic dispersion, centrifugal elutriation, and surgical dissection). The increasing pattern of expression in spermatogonia, spermatocyte, and spermatid qualitatively matches the RNA-seq data from our study presented in **Fig. 2**. Error bars reflect standard error of the mean. (**C**) Immunofluorescence images of wildtype male epididymis containing maturing spermatozoa. CSMD1 protein (red) is highly expressed in the epithelial stereocilia of the epididymis (white arrowheads), but not on the spermatozoa (white dashed arrows), consistent with RNA expression data. Source data are provided as a Source Data file.

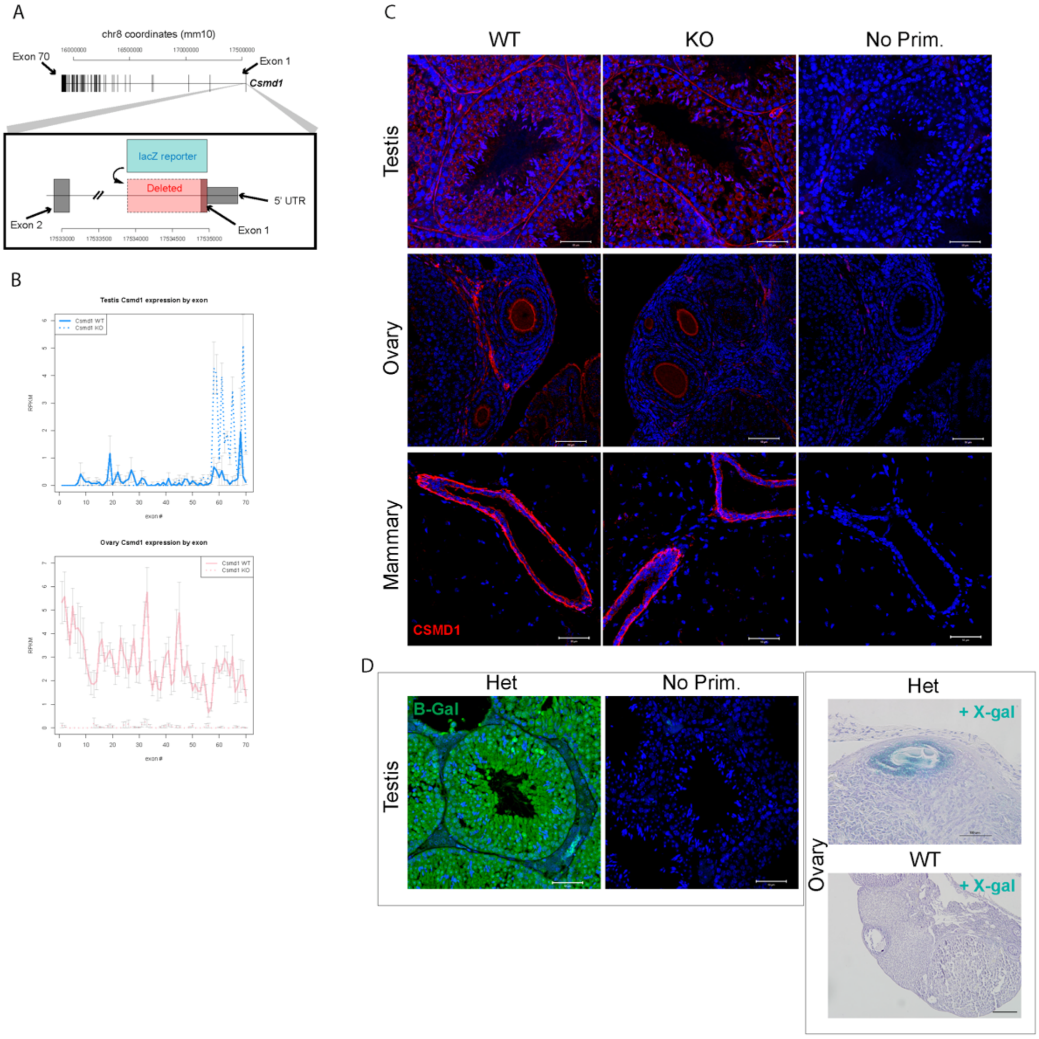

Supplementary Fig. 4. Documenting the effect of *Csmd1*$^{tm1Lex/tm1Lex}$ on RNA and protein expression in gonads and mammaries.
(**A**) Schematic of the mouse *Csmd1* gene model which contains 70 exons spanning 1.6 Mb. Note that human orthologous mutations from our rare variant study are clustered closer to Exon 1. In the *Csmd1*$^{tm1Lex/tm1Lex}$ construct (inset), 1,086 bp encompassing the start codon in Exon 1 and a portion of Intron 1 is deleted and replaced with a lacZ/Neomycin cassette[4]. (**B**) *Csmd1* testis and ovary mRNA expression across Exons 1-70 in Csmd1 wildtype (solid lines) and KO (dotted lines) mice. In ovaries, KO read counts relative to wildtype are suppressed across all 70 exons. In testes, knockout read counts relative to wildtype are broadly suppressed across exons 1-57 and upregulated from exons 58-70. The amino acid coding portion of these upregulated exons range in size from 45 bp to 180 bp. Residual expression of Exon 70 was previously observed in *Csmd1* knockout brain tissue[5]. Error bars reflect standard error of the mean. (**C**) CSMD1 IF in testis, ovary, and mammary. CSMD1 signal is visible in both wildtype and KO mice. (**D**) Orthogonal validation of CSMD1 protein localization. Left: Cross section of *Csmd1* heterozygous testis showing positive signal for anti-Beta-galactosidase reporter cassette not detected in no-primary control. Right: Cross section of *Csmd1* heterozygous vs. wildtype ovary with X-gal treatment. As expected we do not detect any positive β-gal signal in *Csmd1* wildtype, which does not carry the lacZ cassette. β-gal expression approximates patterns of wildtype α-CSMD1 IF expression shown in **Fig. 2.** *Csmd1* paralogs (*Csmd2*, *Csmd3*) are located on different chromosomes than the reporter cassette. Source data are provided as a Source Data file.

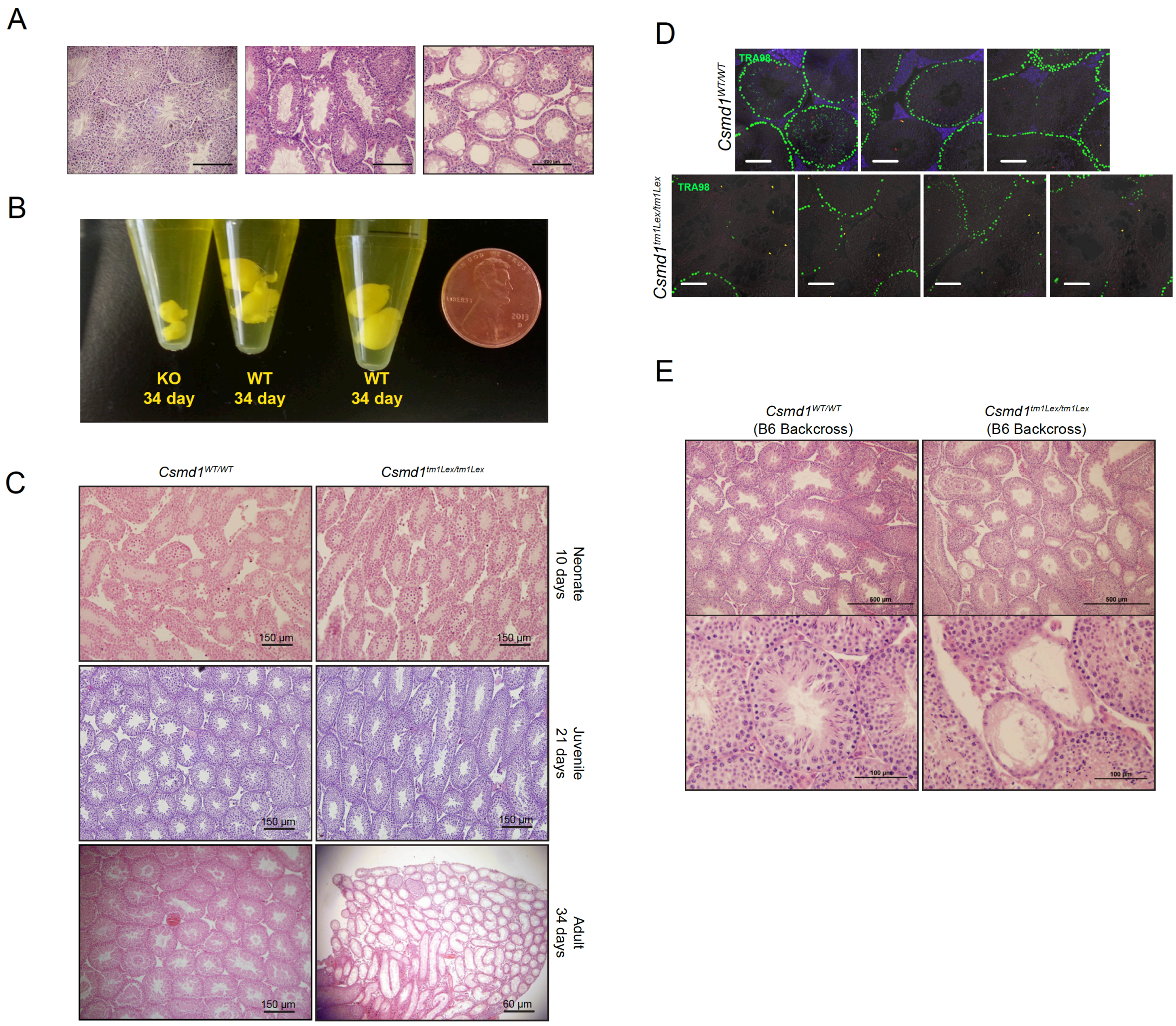

Supplementary Fig. 5. Detailed pathologic characterization of *Csmd1* knockout testes.
(**A**) *Csmd1* KO testes show heterogeneity in timing, location, and severity of histopathology. Representative examples of tubules scored for quantification in **Fig. 3E**. Testes demonstrating no damage ("none", left) show minimal loss of germ cells and tubules display spatial organization of spermatogenesis. Testes demonstrating "mild" (middle) damage display noticeable loss of germ cells, but many tubules maintain some degree of spatial organization. Testes demonstrating "severe" (right) damage have lost many or all germ cells and/or display severe loss of spatial organization. (**B**) Gross morphology of *Csmd1* wildtype and knockout testes. Left to right: *Csmd1* knockout, wildtype, and wildtype littermates sacrificed at 34 days of age. Histology for these individuals is presented in the bottom row of Panel (**C**). (**C**) No evidence of testes degeneration prior to sexual maturity (approx. 30 days). Histological sections of *Csmd1* wildtype (and knockout during the neonatal (10 days), juvenile (21 days) and adult (34 days) time periods. Neonate and juvenile samples are not yet sexually mature evidenced by the absence of mature elongated spermatids. (**D**) Germ-cell marker TRA98 is underexpressed in knockout testes. IF for antibody against TRA98 (green) in *Csmd1* wildtype (upper panels) and knockout (lower panels) littermate testes. The number of TRA98-positive cells ($mean_{wt}$ =144, $mean_{ko}$ = 60.3), is significantly lower in KO tubules after normalizing for total cell number (Poisson regression p-value < 2 x 10^{-16}). All scale bars correspond to 350 µm. (**E**) Degeneration is recapitulated in F5 backcrossed knockout testes. We serially backcrossed the *Csmd1*$^{tm1Lex/tm1Lex}$ allele onto a homogenous C57BL/6 background for 5 generations and examined histological sections of a set of young (< 2 months) wildtype and knockout littermates. Two out of two KO samples showed evidence of moderate to severe degeneration in a subset of the tubules.

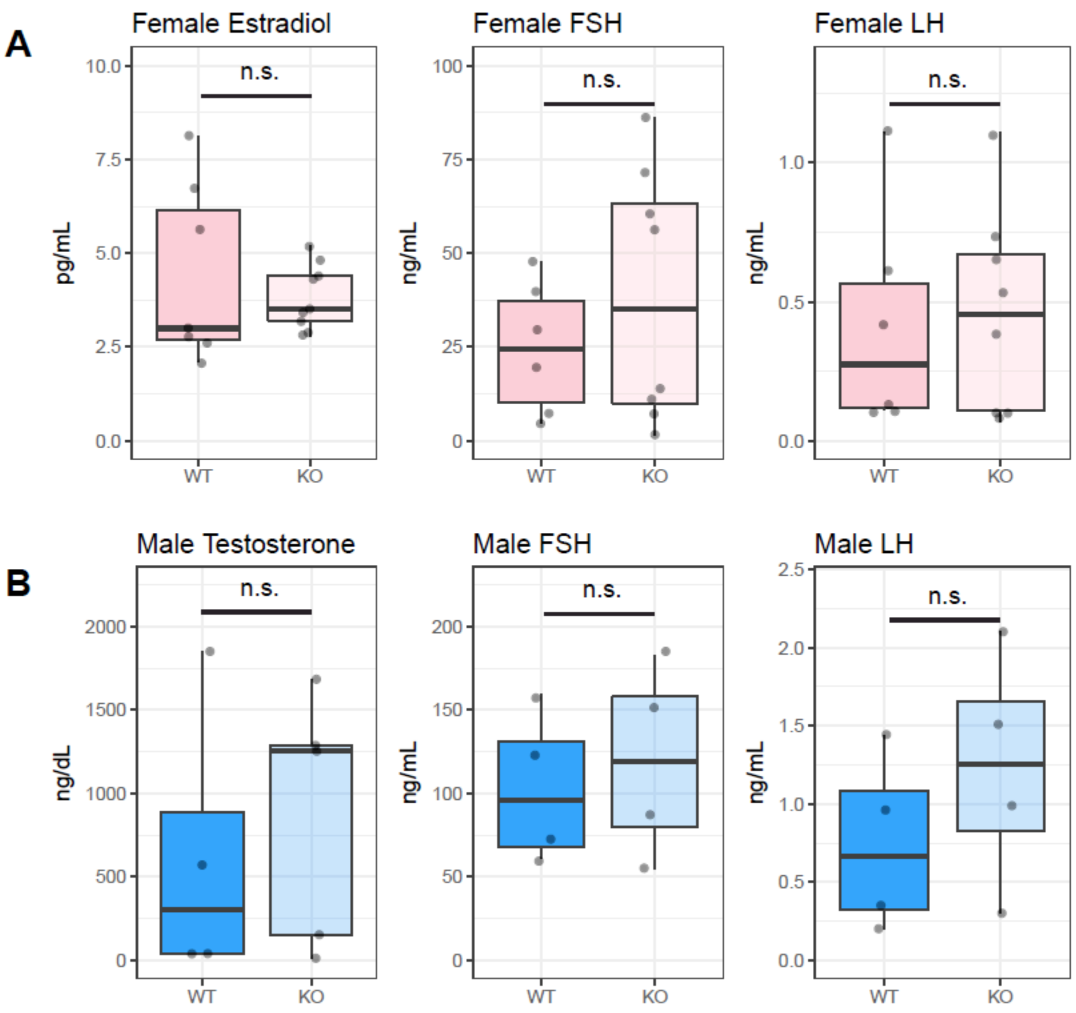

Supplementary Fig. 6. Male and female sex hormone measurements.
Boxplots depicting sex hormone measurements along the pituitary-gonadal axis in (**A**) females (estradiol, FSH, and LH) and (**B**) males (testosterone, FSH, and LH). No significant differences in hormone levels were detected between wildtypes and knockouts (two-tailed *t*-test p-value > 0.05). Center line = median; hinges = upper and lower quartiles; whiskers = 1.5 x interquartile range. Source data are provided as a Source Data file.

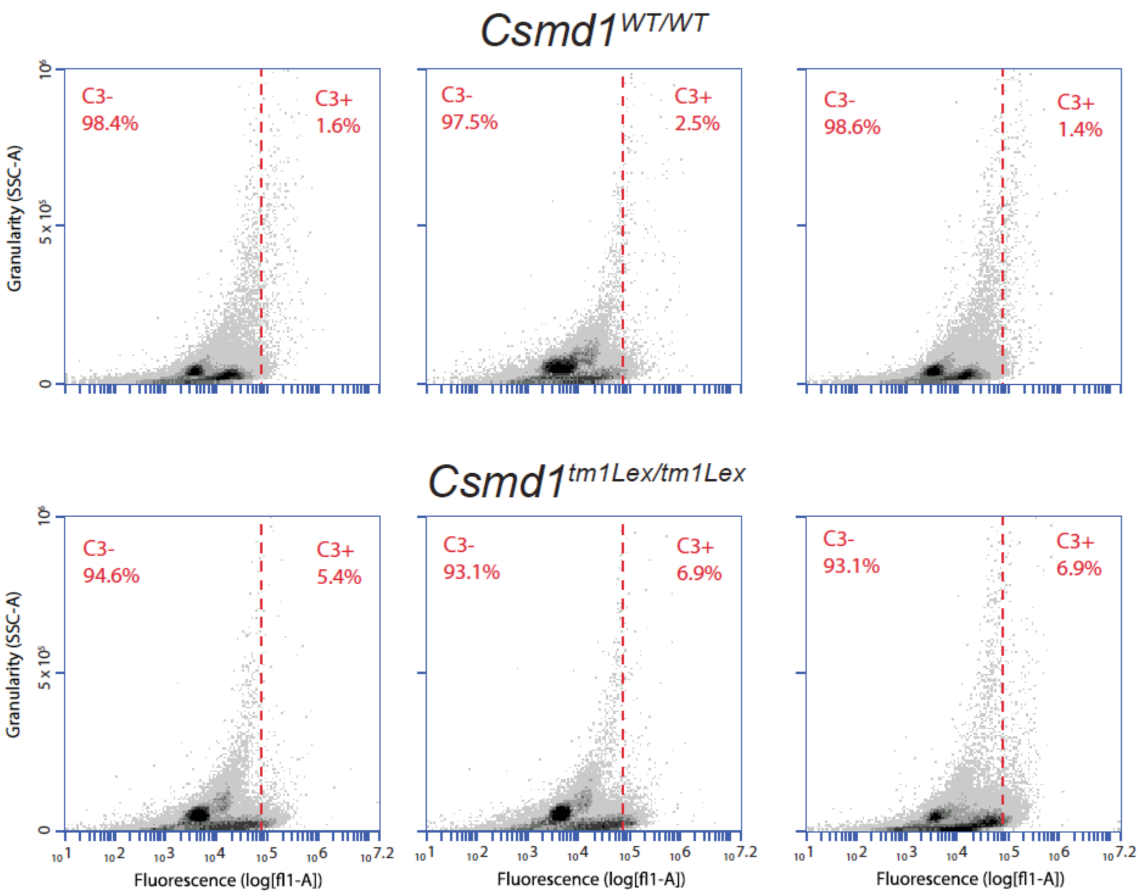

Supplementary Fig. 7. C3 deposition in *Csmd1* wildtype versus knockout testes.
FACS output depicting total C3 deposition on *Csmd1* wildtype and knockout testes collected with identical gating parameters.

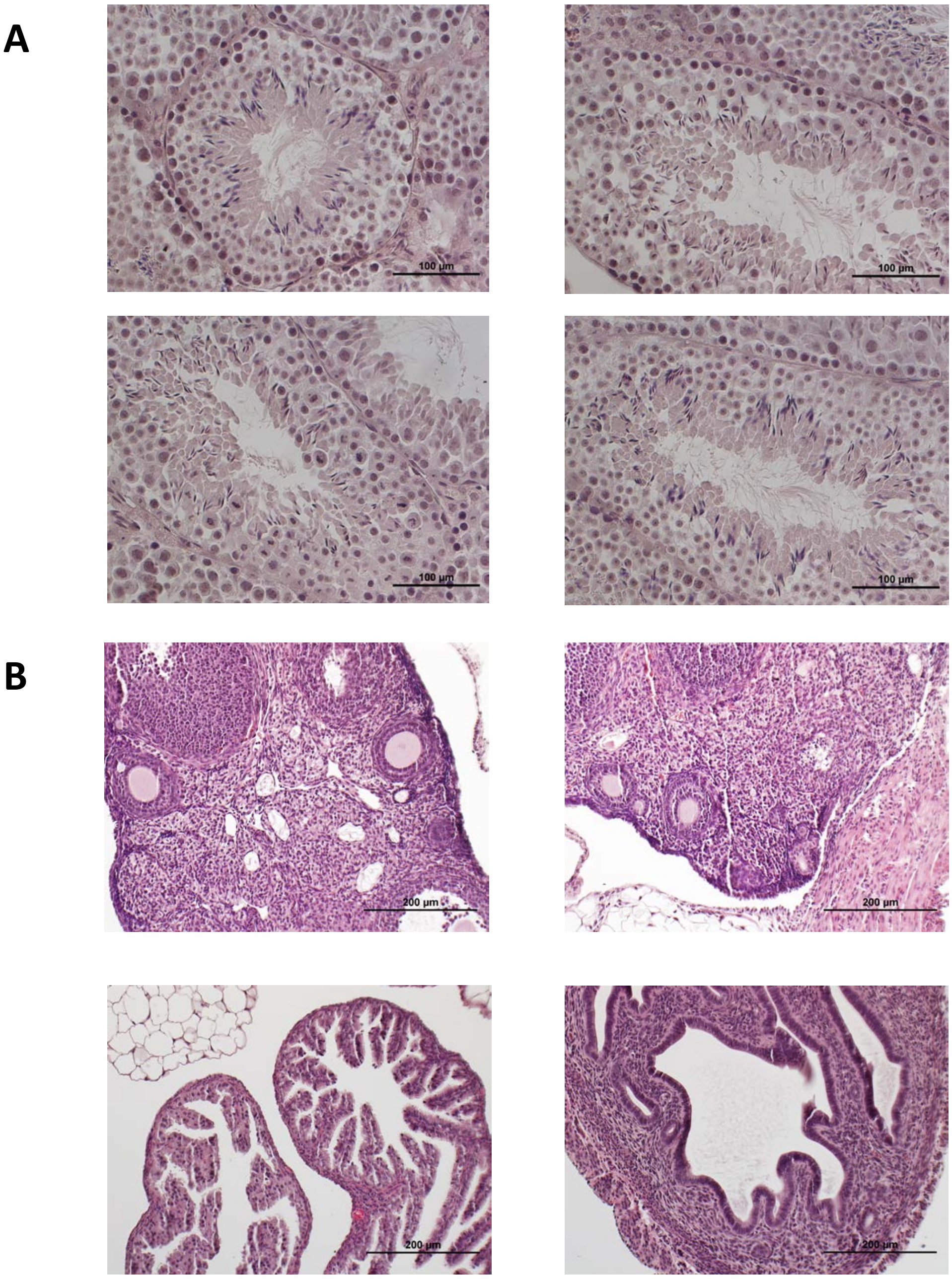

Supplementary Fig. 8. C3$^{-/-}$ gonads are histologically normal.
(**A**) Example H&E testis histology from 39 day old *C3*$^{-/-}$ male. Testes are fixed in PFA. (**B**) Example H&E ovary (top) and oviducts (bottom) from *C3*$^{-/-}$ female.

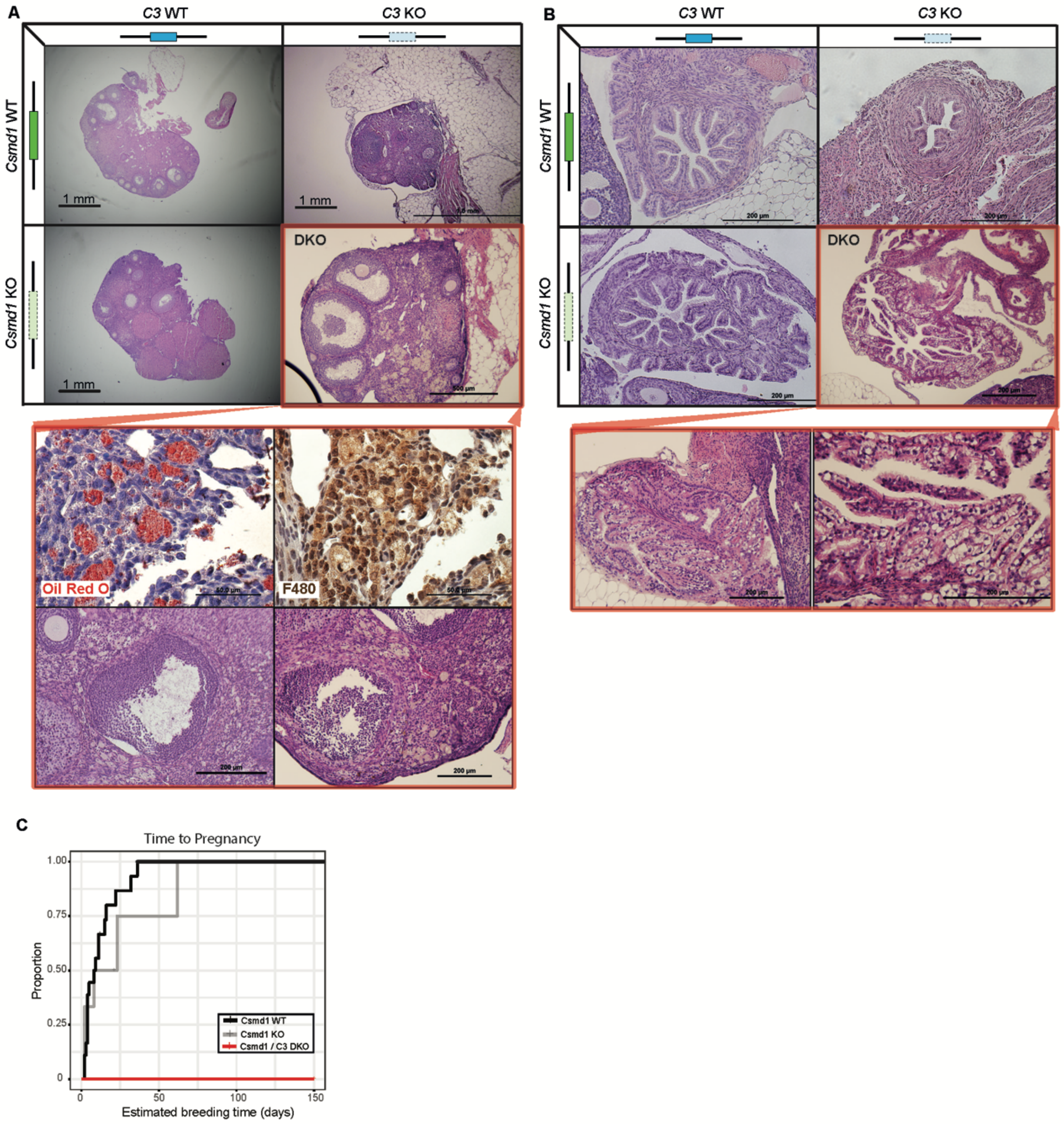

Supplementary Fig. 9. Complement deficiency unmasks severe morphological derangement in *Csmd1* knockout ovaries.

(**A**) Example H&E ovarian histology of *C3* and *Csmd1* wildtype/knockout combinations. Closer inspection of *C3/Csmd1* double knockout ovaries shows profound inflammatory changes of the of the stroma with associated foam cell macrophages which stain positively for Oil Red O and F4/80. Follicle shape is grossly deformed. (**B**) H&E histology of *C3/Csmd1* double knockout oviducts showing loss of cellularity of the epithelial mucosa. (**C**) Timed breeding compares mating success among *Csmd1* wildtype (black), *Csmd1* knockout (grey), and *C3/Csmd1* double knockout (red) females. *C3/Csmd1* double knockout females produced zero offspring after > 5 months of breeding. A subset of *Csmd1* WT and KO breeding data are replotted from **Fig. 4** for ease of comparison. Source data are provided as a Source Data file.

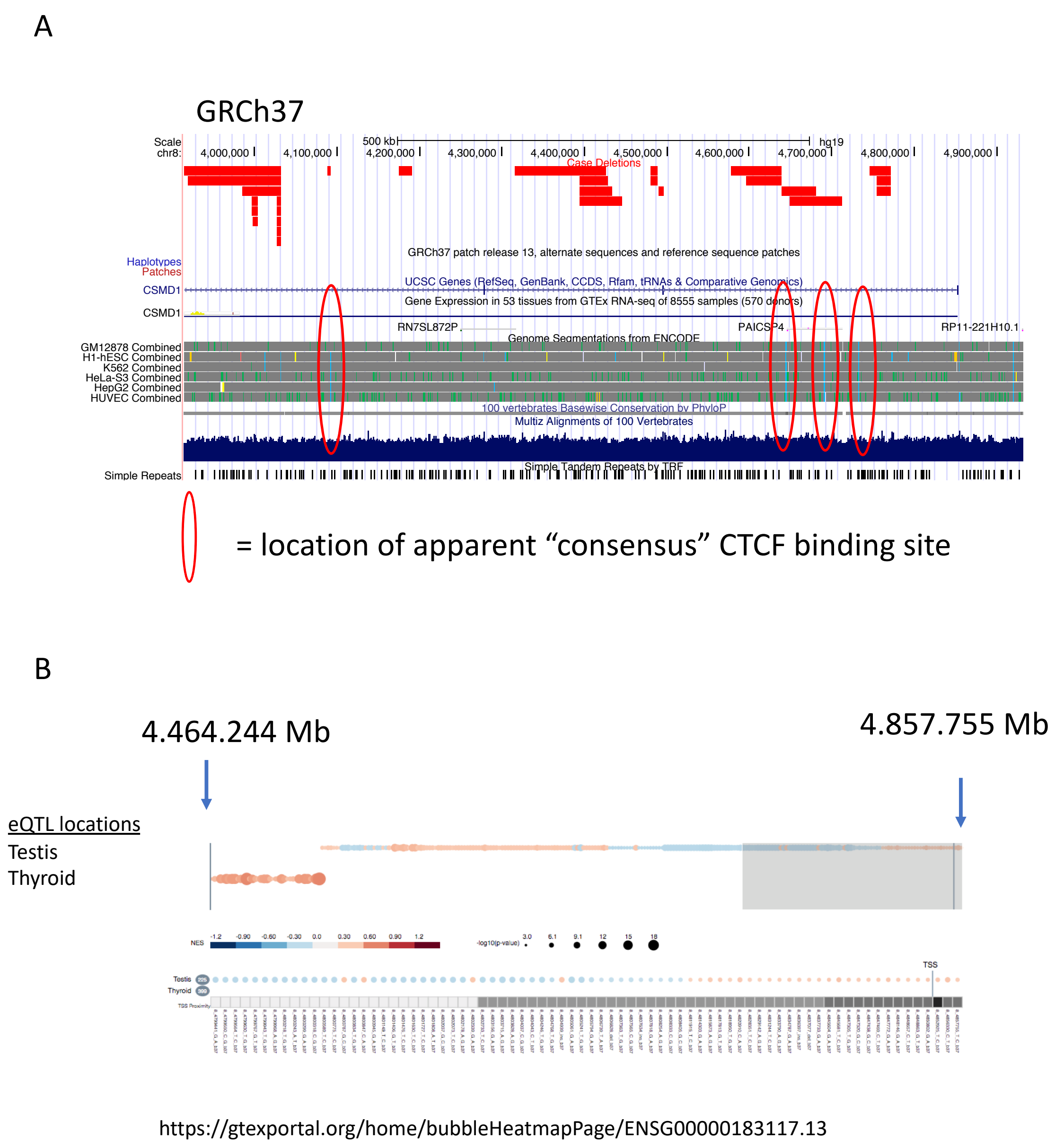

Supplementary Fig. 10. Functional genomic annotations of *CSMD1* indicate regulatory power of introns 1 and 2.

(**A**) Screenshot of the UCSC Genome Browser displaying the "Genome Segmentations" ENCODE track for the first three introns of CSMD1 (GRCh37 assembly). Four CTCF elements have been annotated across all (or most) of the 6 human cell lines used to generate the track. Shown at the top, as a custom track, are the disease-associated *CSMD1* deletions reported in this study (track name: "Case Deletions"). (**B**) A screenshot of the GTEx portal eQTL viewer, showing the location of all *CSMD1* eQTLs detected by the study. The region spans a 393 kb window of the gene at the 5' end, involving introns 1 and 2. Each "bubble" represent the location of an eQTL; as shown in the legend, the color of the bubble reflects its normalized effect size (NES) while the size of the bubble reflects the p-value of the test of the hypothesis that each SNP is not associated with CSMD1 expression. The URL for these data is https://gtexportal.org/home/bubbleHeatmapPage/ENSG00000183117.13.

Supplementary Tables

Supplementary Table 1. Rare CNVs associated with gonadal function in WHI-SHARe and GEMINI. We performed a gene-based genome-wide association study in the WHI-SHARe cohort to identify rare CNVs associated with female gonadal dysfunction (Stage I) and attempted replication in males (Stage II).

All genes with nominal association p-values < 0.05 in Stage I, and the analogous values for Stage II are listed; a value of "N/A" indicates that no CNVs were observed at the locus.

Of note, biallelic knockout of *Mcph1* was reported to cause infertility in male and female mice[6].

chr	gene	Female nominal p-value	Female corrected p-value	Male replication p-value	Female replication p-value
1	PRAMEF8	0.0018	0.1944	N/A	N/A
1	PRAMEF15	0.0018	0.1944	N/A	N/A
1	PRAMEF9	0.0018	0.1944	N/A	N/A
1	PRAMEF14	0.0018	0.1944	N/A	N/A
8	CSMD1	0.0004	0.0148	0.0031	0.0004
8	MCPH1	0.0048	0.4169	N/A	N/A

Supplementary Table 2. Genotype-phenotype information for a subset of CSMD1 5'-deletion early idiopathic menopause cases in the WHI.

	CASE 1	CASE 2	CASE 3	CASE 4	Other cases [1]	Controls [2]
CSMD1 5'-deletion variant (hg38 chr8)	g.(4458532_4569017)del	g.(4622470_4631362)del	g.(4622470_4630524)del	g.(4622470_4630524)del	—	—
Size (kb)	110.5	8.9	8.1	8.1		
VEP classification	Regulatory region variant, TF binding site variant	Regulatory region variant	Regulatory region variant	Regulatory region variant	—	—
Ever smoked	—	Yes	Yes	-	51.3% Yes	47.7% Yes
Age at menopause	35	35	30	37	35	49
Age at menarche	13	16	12	11	13	13
Lifetime pregnancies	2	1	1	3	3	3
Breastfed >= 1 mo.	—	—	Yes	—	50.7% Yes	50.7% Yes

[1] Mean proportion or median value in non-*CSMD1*-5’-deletion cases.

[2] Mean proportion or median value in controls

Supplementary Table 3. Results of breeding *Csmd1*$^{-/-}$ *; C3*$^{-/-}$ double knockouts.

Genotype	Sex	Mated with	Duration of mating	Litter born	1st litter Size	2nd litter Size	3rd litter Size
Csmd-/- C3-/-	M	Csmd-/- C3-/-	5 months	No			
Csmd-/- C3-/-	M	Csmd-/- C3-/-	5 months	No			
Csmd-/- C3-/-	F	Csmd-/- C3-/-	5 months	No			
Csmd-/- C3-/-	F	Csmd-/- C3-/-	5 months	No			
Csmd-/- C3-/-	M	Csmd-/- C3-/-	5 months	No			
Csmd-/- C3-/-	M	Csmd-/- C3-/-	5 months	No			
Csmd-/- C3-/-	F	Csmd-/- C3-/-	5 months	No			
Csmd-/- C3-/-	F	Csmd-/- C3-/-	5 months	No			
Csmd-/- C3-/-	M	WT (2)	7 months	No			
Csmd-/- C3-/-	M	WT(1)	5 months	Yes	3	4	3
Csmd-/- C3-/-	M	WT(1)	5 months	Yes	2	7	5
Csmd-/- C3-/-	F	WT(1)	5 months	Yes	4	6	
Csmd-/- C3-/-	M	WT(1)	3 months	No			
Csmd-/- C3-/-	M	WT(1)	3 months	Euthanized	died in delivery		
Csmd-/- C3-/-	M	WT(1)	3 months	No			
Csmd-/- C3-/-	F	WT(1)	3 months	No			
Csmd-/- C3-/-	F	WT(1)	3 months	Found Dead			
Csmd-/- C3-/-	F	WT(1)	3 months	No			
Csmd-/- C3-/-	F	WT(1)	3 months	No			

Supplementary References

1. Hammoud, S. S. *et al.* Chromatin and transcription transitions of mammalian adult germline stem cells and spermatogenesis. *Cell Stem Cell* (2014). doi:10.1016/j.stem.2014.04.006

2. Gan, H. *et al.* Integrative proteomic and transcriptomic analyses reveal multiple post-transcriptional regulatory mechanisms of mouse spermatogenesis. *Mol. Cell. Proteomics* (2013). doi:10.1074/mcp.M112.020123

3. Soumillon, M. *et al.* Cellular Source and Mechanisms of High Transcriptome Complexity in the Mammalian Testis. *Cell Rep.* (2013). doi:10.1016/j.celrep.2013.05.031

4. Friddle, C. J. *et al.* High-throughput mouse knockouts provide a functional analysis of the genome. in *Cold Spring Harbor Symposia on Quantitative Biology* (2003).

5. Distler, M. G., Opal, M. D., Dulawa, S. C. & Palmer, A. A. Assessment of Behaviors Modeling Aspects of Schizophrenia in Csmd1 Mutant Mice. *PLoS One* (2012). doi:10.1371/journal.pone.0051235

6. White, J. K. *et al.* Genome-wide Generation and Systematic Phenotyping of Knockout Mice Reveals New Roles for Many Genes. *Cell* **154**, 452–64 (2013).
