# Supplementary material for: Rare mutations in the complement regulatory gene CSMD1 are associated with male and female infertility
Source: Nat Commun. 2019 Oct 11;10:4626. doi: 10.1038/s41467-019-12522-w (PMC6789153; doi:10.1038/s41467-019-12522-w)
Supplement: Supplementary file 5 — Reporting Summary [file 41467_2019_12522_MOESM5_ESM.pdf]

nature research

Corresponding author(s): Don Conrad

Last updated by author(s): Sep 3, 2019

Reporting Summary

Nature Research wishes to improve the reproducibility of the work that we publish. This form provides structure for consistency and transparency in reporting. For further information on Nature Research policies, see Authors & Referees and the Editorial Policy Checklist.

Statistics

For all statistical analyses, confirm that the following items are present in the figure legend, table legend, main text, or Methods section.

n/a	Confirmed	
☐	☒	The exact sample size (n) for each experimental group/condition, given as a discrete number and unit of measurement
☐	☒	A statement on whether measurements were taken from distinct samples or whether the same sample was measured repeatedly
☐	☒	The statistical test(s) used AND whether they are one- or two-sided<br>*Only common tests should be described solely by name; describe more complex techniques in the Methods section.*
☐	☒	A description of all covariates tested
☐	☒	A description of any assumptions or corrections, such as tests of normality and adjustment for multiple comparisons
☐	☒	A full description of the statistical parameters including central tendency (e.g. means) or other basic estimates (e.g. regression coefficient) AND variation (e.g. standard deviation) or associated estimates of uncertainty (e.g. confidence intervals)
☐	☒	For null hypothesis testing, the test statistic (e.g. F, t, r) with confidence intervals, effect sizes, degrees of freedom and P value noted<br>*Give P values as exact values whenever suitable.*
☒	☐	For Bayesian analysis, information on the choice of priors and Markov chain Monte Carlo settings
☒	☐	For hierarchical and complex designs, identification of the appropriate level for tests and full reporting of outcomes
☐	☒	Estimates of effect sizes (e.g. Cohen's d, Pearson's r), indicating how they were calculated

Our web collection on statistics for biologists contains articles on many of the points above.

Software and code

Policy information about availability of computer code

Data collection	CopyCaller (TaqMan), FlowJo (FACS), Zen software (microscopy), Olympus DP software (microscopy). Described in detail in the methods section.
Data analysis	R statistical environment, Sequence Kernel Association Test (SKAT), Combined Annotation Dependent Depletion (CADD), Affy6CNV, Birdsuite, PennCNV, TopHat2, Probablistic Estimation of Expression Residuals (PEER), poissonSeq, ImageJ, PLINK, Adobe Photoshop. Described in detail in the methods section.

For manuscripts utilizing custom algorithms or software that are central to the research but not yet described in published literature, software must be made available to editors/reviewers. We strongly encourage code deposition in a community repository (e.g. GitHub). See the Nature Research guidelines for submitting code & software for further information.

Data

Policy information about availability of data

All manuscripts must include a data availability statement. This statement should provide the following information, where applicable:

- Accession codes, unique identifiers, or web links for publicly available datasets
- A list of figures that have associated raw data
- A description of any restrictions on data availability

Human sample data are from WHI-SHARe (dbGaP Study Accession phs000386.v7.p3 [https://www.ncbi.nlm.nih.gov/projects/gap/cgi-bin/study.cgi?study_id=phs000386.v7.p3]), WHISP (dbGaP Study Accession phs000281.v6.p3 [https://www.ncbi.nlm.nih.gov/projects/gap/cgi-bin/study.cgi?study_id=phs000281.v6.p3]), UK Biobank (www.ukbiobank.ac.uk), and GEMINI (www.gemini.conradlab.org) and can be obtained upon application. GTEx data were viewed through www.gtexportal.org and ENCODE data were viewed in the UCSC genome browser (genome.ucsc.edu). RNA-seq data generated in this study have been submitted to the Gene Expression Omnibus (accession number GSE136769 [https://www.ncbi.nlm.nih.gov/geo/query/acc.cgi?acc=GSE136769]). A reporting summary for this Article is available as a Supplementary Information file. The source data underlying Figures 1-5 and Supplementary Figures 1, 3, 6, and 9 are provided as a Source Data file. Additional information is available upon request.

Field-specific reporting

Please select the one below that is the best fit for your research. If you are not sure, read the appropriate sections before making your selection.

☒ Life sciences ☐ Behavioural & social sciences ☐ Ecological, evolutionary & environmental sciences

For a reference copy of the document with all sections, see nature.com/documents/nr-reporting-summary-flat.pdf

Life sciences study design

All studies must disclose on these points even when the disclosure is negative.

Sample size	Discovery sample size based on the Women's Health Initiative SNP Health Association Resource (WHI-SHARe). We performed no bespoke sample size calculation for the discovery phase. Replication sample size based on UK Biobank cohort study. Sample size was greater than or comparable to analogous studies previously published from our group and others. Sample size was adequate due to implicit assumptions made about the effect-size/allele frequency architecture of the phenotype as described in the main text and methods. A corollary to this approach is that we are not adequately powered, nor do we claim, to detect common associated variants of small effect size.
Data exclusions	Cases were excluded based on published risk factors that confound the phenotype or included in the statistical model as covariates. Stringent call and sample QC were performed based on previously published studies from our group and others.
Replication	We replicated our discovery association with >60,000 UK Biobank samples. All attempts at replication were successful.
Randomization	No randomization (case-control study).
Blinding	All genotypes were blinded with respect to participant phenotype status.

Reporting for specific materials, systems and methods

We require information from authors about some types of materials, experimental systems and methods used in many studies. Here, indicate whether each material, system or method listed is relevant to your study. If you are not sure if a list item applies to your research, read the appropriate section before selecting a response.

Materials & experimental systems

n/a	Involved in the study	
☐	☒	Antibodies
☒	☐	Eukaryotic cell lines
☒	☐	Palaeontology
☐	☒	Animals and other organisms
☐	☒	Human research participants
☒	☐	Clinical data

Methods

n/a	Involved in the study	
☒	☐	ChIP-seq
☐	☒	Flow cytometry
☒	☐	MRI-based neuroimaging

Antibodies

Antibodies used	CSMD1: goat anti-CSMD1 N20 (Santa Cruz Biotechnology sc-68280 1:100); MVH: rabbit anti-mouse vasa homolog (Abcam 13840 1:1000); donkey anti-goat CF594 (Biotium 1:300); donkey anti-rabbit Alexa488 (Life Technologies 1:300); rabbit anti-beta-gal (Cappel 1:333); F480: rat F4/80 BM8 (Santa Cruz Biotechnology sc-52664 1:50); donkey anti-rat Alexa488 (Life Technologies, 1:300); C3: rabbit anti-C3 (Abcam 200999 1:2000), goat anti-rabbit Alexa568 (Life Technologies); biotin-coupled horse anti-goat (Vector Laboratories BA-9500 1:200); C3 (B9): mouse anti-C3 (Santa Cruz Biotechnologies sc-28294 1:100).
Validation	CSMD1: recommended for mouse IF and IHC per manufacturer's datasheet; MVH: recommended for mouse IF per manufacturer's datasheet; F480: recommended for mouse IF and IHC per manufacturer's datasheet; C3: recommended for mouse IHC per manufacturer's datasheet; C3 (B9): recommended for mouse IF and IHC per manufacturer's datasheet.

Animals and other organisms

Policy information about studies involving animals; ARRIVE guidelines recommended for reporting animal research

Laboratory animals	Mus musculus, B6;129S5 (described in detail in methods and previously by https://doi.org/10.1371/journal.pone.0051235), males and females, ages postnatal day 1 to > 300.
Wild animals	N/A

Field-collected samples	N/A
Ethics oversight	All animal experiments were performed in compliance with the regulations of the Animal Studies Committee at Washington University in St. Louis under protocols #20120244 and #20160089.

Note that full information on the approval of the study protocol must also be provided in the manuscript.

Human research participants

Policy information about studies involving human research participants

Population characteristics	A detailed description of study participants is available in the methods. Detailed description of WHI study participants is available at https://www.ncbi.nlm.nih.gov/projects/gap/cgi-bin/study.cgi?study_id=phs000386.v7.p3. Detailed description of UK Biobank study participants is available at http://biobank.ctsu.ox.ac.uk/crystal/label.cgi.
Recruitment	N/A
Ethics oversight	All human genetic studies were reviewed and approved by the Institutional Review Board of Washington University in St. Louis, under protocol numbers #201107177 and #201109261.

Note that full information on the approval of the study protocol must also be provided in the manuscript.

Flow Cytometry

Plots

Confirm that:

☒ The axis labels state the marker and fluorochrome used (e.g. CD4-FITC).

☒ The axis scales are clearly visible. Include numbers along axes only for bottom left plot of group (a 'group' is an analysis of identical markers).

☒ All plots are contour plots with outliers or pseudocolor plots.

☒ A numerical value for number of cells or percentage (with statistics) is provided.

Methodology

Sample preparation	Detailed description of sample preparation is available in the methods and published previously (e.g., doi:10.1002/cyto.a.20129). Briefly, sexually mature (40 ± 1 days old), wildtype male mice were sacrificed, and their testes were decapsulated and homogenized in a 1X MEM solution (Gibco 11430-030) containing 120 U/mL Type I Collagenase (Worthington Biochemical LS004194) and 1 mg/mL DNAse I (Roche 10104159001), and agitated for 15 minutes. 1X MEM was replaced and added with 50 mg/mL Trypsin (Worthington Biochemical 54J15037) and 1 mg/mL DNAse I and agitated for 15 minutes, then mechanically homogenised for 3 minutes. 50 mg/mL Trypsin and 1 mg/mL DNAse I were added and agitated again for 15 minutes. We added 0.4 mL heat inactivated Fetal Bovine Serum (Sigma F1051), 5 µL Hoescht 33342 (Life Technologies H3570), and 1 mg/mL DNAse I, and agitated for 15 minutes. Individual cells were dissociated by pipetting sequentially through two 40 µm cell strainers (Falcon 352340). For each individual mouse, one dissociated testis was used for whole tissue RNA extraction and sequencing, and the other testis was used for germ cell purification, RNA extraction, and sequencing. All dissociation steps were performed at 33°C.
Instrument	Custom Beckman Coulter MoFlo at the Washington University Siteman Flow Cytometry Core (additional information available at https://siteman.wustl.edu/research/shared-resources-cores/siteman-flow-cytometry-sfc/siteman-flow-cytometry-core-cell-sorters/).
Software	FlowJo software package
Cell population abundance	Sorted cell populations from this protocol are highly enriched for germ cells of interest as show previously by our group and others (e.g., doi:10.1002/cyto.a.20129; doi: 10.3791/55913). We performed RNA-seq on sorted germ cell populations and show strong enrichment for transcripts corresponding to cell type of interest.
Gating strategy	Using the FSC/SSC gating, debris was removed by gating on the main cell population. Specific representative examples of selected gates are provided in Supplementary Information.

☒ Tick this box to confirm that a figure exemplifying the gating strategy is provided in the Supplementary Information.
