# Supplementary material for: Rare mutations in the complement regulatory gene CSMD1 are associated with male and female infertility
Source: Nat Commun. 2019 Oct 11;10:4626. doi: 10.1038/s41467-019-12522-w (PMC6789153; doi:10.1038/s41467-019-12522-w)
Supplement: Supplementary file 7 — Description of Additional Supplementary Files [file 41467_2019_12522_MOESM7_ESM.pdf]

Title: Supplementary Data 1.
Description: The full set of CNV calls and inferred POI case/control status that we generated from the WHI SHARe cohort.

Title: Supplementary Data 2.
Description: All deletions detected in introns 1-3 from SHARe, the azoospermia cohort, and UK Biobank, along with case/control status of each deletion carrier.

Title: Supplementary Data 3.
Description: Lookup table linking sample, genotype, tissue, and RPKM information.
